# Changemaker: study protocol for sustainable adolescent health interventions to reduce non-communicable disease risks in urban Burkina Faso, Kenya and Tanzania – a hybrid type 2 cluster-randomised controlled trial

**DOI:** 10.1136/bmjopen-2026-123282

**Published:** 2026-10-28

**Authors:** Raissa Sorgho, Sachin Shinde, Pilvikki Absetz, Helle Mölsted Alvesson, Joseph Ochieng Amoke, Anu Linnansaari, Linda B Mlunde, Abdu Mohiddin, Erick M O Muok, Alice Achieng Ojwang, Mary Mwanyika Sando, Ali Sié, Abdramane Bassiahi Soura, Bruno Sunguya, Marleen Temmerman, Amani Tinkasimile, Arthur Wamani, Changemaker Consortium, Marleen Temmerman, Kristi Sidney Annerstedt, Ina Danquah

**Affiliations:** 1 Transdisciplinary Research Area "Technology and Innovation for Sustainable Futures" and Center for Development Research (ZEF), University of Bonn, Bonn, Germany; 2 Global Health and Population, Harvard T.H. Chan School of Public Health, Boston, Massachusetts, USA; 3 Tampere University, Tampere, Finland; 4 Global Public Health, Karolinska Institutet, Stockholm, Sweden; 5 Department of Agriculture, Livestock and Fisheries (DALF), County Government of Kisumu, Kisumu, Kenya; 6 School of Public Health and Social Sciences, Muhimbili University of Health and Allied Sciences (MUHAS), Dar es Salaam, Tanzania; 7 Aga Khan University, Nairobi, Kenya; 8 Kenya Medical Research Institute (KEMRI), Kisumu, Kenya; 9 Technical University of Kenya, Nairobi, Kenya; 10 Africa Academy for Public Health (AAPH), Dar es Salaam, United Republic of Tanzania; 11 Centre de Recherche en Santé de Nouna (CRSN), Nouna, Burkina Faso; 12 Institut Supérieur des Sciences de la Population (ISSP), Joseph Ki-Zerbo University of Ouagadougou, Ouagadougou, Burkina Faso; 13 Centre of Excellence Women and Child Health, Aga Khan University, Nairobi, Kenya; 14 Food Security for Peace and Nutrition (FSPN), Nairobi, Kenya; 15 Heidelberg Institute of Global Health (HIGH), Medical Faculty and University Hospital, Heidelberg University, Heidelberg, Germany

**Keywords:** Adolescent, Noncommunicable Diseases, Schools, PUBLIC HEALTH, Primary Prevention

## Abstract

**Introduction:**

Adolescent obesity is a growing global health concern that significantly contributes to adult multimorbidity and the rising burden of non-communicable diseases (NCDs). Adolescence presents a critical window for shaping lifelong health behaviours that can prevent these outcomes. Effective health promotion during this stage can play a pivotal role in the primary prevention of NCDs. In low- and middle-income countries, urban adolescents encounter a complex interplay of risk factors, including unhealthy diets, physical inactivity and limited access to supportive health environments. However, evidence on designing and sustainably implementing multi-component, adolescent-centred health promotion and prevention interventions in these settings remains limited. This study aims to evaluate the implementation and effectiveness of a multicomponent intervention focusing on the primary prevention of NCD risks among adolescents.

**Methods and analysis:**

After a co-design process, we will conduct three cluster-randomised controlled trials in secondary schools in three sub-Saharan African cities: Ouagadougou (Burkina Faso), Kisumu (Kenya) and Dar es Salaam (Tanzania). The study will include 2280 participants aged 12–16 years (190 students per school across four schools at each site). Over one academic year, a four-component intervention will be implemented: (1) establishment of school gardens and organic waste composting; (2) integration of sustainable health education modules into classes; (3) delivery of health talks using motivational strategies and (4) creating mass media campaigns promoting healthy lifestyles. The primary effectiveness outcomes for health and nutrition constitute mid-upper arm circumference z-score and haemoglobin concentration, respectively. Implementation outcomes will be assessed through a process evaluation examining participation and emotional and cognitive engagement (primary implementation outcomes), along with acceptability, adoption, feasibility, fidelity, reach and sustainability (secondary implementation outcomes). We will also evaluate the implementation costs of the intervention.

**Ethics and dissemination:**

The Institutional Review Board (IRB) of University Hospital and Medical Faculty, University of Bonn (UoB) reviewed and approved the Changemaker study protocol and all its annexes in July 2024 (reference: 2024-265 BO). This serves as the principal approval from one of the coordinating institutions. In addition, the study protocol, its processes, and tools were reviewed and approved by local IRBs of the participating institutions. All participants will provide informed written consent or assent. Data will be pseudonymised and stored on secure UoB servers. Results will be disseminated via peer-reviewed publications, conferences and participant summaries.

**Trial registration number:**

DRKS00034129.

**STRENGTHS AND LIMITATIONS OF THIS STUDY**

- The study integrates health promotion with environmental sustainability to target key behavioural and environmental drivers of adolescent health.
- The hybrid type 2 cluster-randomised controlled trial design allows to simultaneously assess implementation outcomes such as feasibility, acceptability, appropriateness and adoption across diverse cultural settings, providing a comprehensive understanding of what works, for whom and under what conditions, supporting the development of scalable and sustainable solutions.
- The trial is grounded in human-centred design, allowing for engagement of adolescents, parents, teachers and multisectoral stakeholders from the outset and throughout all stages of the study.
- Blinding participants, investigators and most study personnel will not be possible due to the nature of behavioural interventions; we will counteract this limitation by blinding some research assistants, data analysts and evaluation teams.

## Introduction

### Background and rationale

 Adolescents aged 10–19 years, who represent approximately 90% of the world’s 1.3 billion, primarily live in low- and middle-income countries (LMICs).^1^ By 2050, sub-Saharan Africa (SSA) is expected to have the largest adolescent population globally, making the region a critical priority for addressing emerging health challenges.^2^ Among these challenges, the rising prevalence of adolescent obesity is a serious public health concern, as it significantly contributes to non-communicable diseases (NCDs) such as cardiovascular disease, type 2 diabetes and certain cancers. NCDs are increasing rapidly in SSA and are projected to surpass communicable diseases as the leading cause of death by 2030.^3^ Many risk factors, including unhealthy dietary habits, low physical activity, sedentary behaviours and tobacco use, develop during adolescence and hinder both mental and physical development.^4 5^

Many countries in SSA face a triple burden of malnutrition, characterised by a persistent burden of undernutrition and micronutrient deficiencies with rising rates of overweight, obesity and diet-related NCDs within the same individuals, households and communities.^6^ Undernutrition in early life has been linked to a higher risk of obesity and other NCDs later in life, underscoring the need for early preventative interventions.^7 8^

The intersection of malnutrition and climate change further exacerbates health and nutrition challenges in the region. Extreme weather events, such as persistent mini-droughts and floods, along with rising temperatures, are significantly reducing crop yields and increasing food insecurity and hunger.^9^ A recent report by the Intergovernmental Panel on Climate Change warns that global food insecurity will rise, with SSA among the most adversely affected regions.^10^ Climate change has also been linked to increasing obesity, particularly in urban areas, through its impacts on food systems, physical activity patterns and environmental conditions.^9^

Urbanisation further intensifies the existing epidemiological landscape, contributing to a growing burden of NCDs through increased sedentary lifestyles, reduced physical activity and greater availability and consumption of ultra-processed, nutrient-poor and high-calorie diets, often accompanied by low fruit and vegetable intake.^11^ The shift towards urbanised diets is strongly associated with unhealthy consumption patterns, which are major contributors to the global burden of diseases, particularly NCDs. The Food and Agriculture Organisation of the United Nations, in its state of Africa report, states that around 282 million people in Africa are undernourished, and urbanisation disproportionately increases the number of lower-income residents, further exacerbating food insecurity in urban populations.^12^

Unhealthy diets are responsible for an estimated 11 million deaths from NCDs, accounting for 22% of all adult deaths, and contributing to nearly half of all deaths and disability in LMICs.^6^ Only 30% of adolescents living in LMICs meet the WHO recommendations for fruit and vegetable intake and physical activity.^13^ The complex interplay of limited resources, urbanised lifestyles and diets, a changing climate, and rising NCD risk factors underscores the urgent need for sustainable health solutions targeting urban adolescents in SSA.

School-based multi-component interventions are critical for addressing obesity and NCD risks among adolescents in LMICs. Such interventions can integrate education, environmental changes and community engagement to comprehensively influence diet and physical activity. Evidence suggests that multi-component interventions, which target multiple underlying contributors to NCD risk, have a greater impact on improving healthy habits than single-component interventions.^14^ However, evidence is mixed on effective strategies to promote health and prevent adolescent overweight and obesity, underscoring the urgent need for innovative and evidence-based solutions.^15^ Testing the effectiveness and cost-effectiveness of multi-component interventions in resource-constrained settings is critical. Furthermore, it is vital to understand what works in different contexts and, if it works, why it works, by evaluating factors such as acceptability, adoption, appropriateness and feasibility.^16^ A hybrid approach that simultaneously evaluates effectiveness, cost-effectiveness and key implementation outcomes is essential to optimise these interventions for broader scale-up.^17^ Such an approach helps to ensure that interventions are not only effective and sustainable but also feasible, culturally appropriate and adaptable to different settings, ultimately guiding successful implementation and long-term impact.

The Changemaker study adopts a sustainable health approach to address interconnected challenges of adolescent obesity, NCD risks and climate change in Ouagadougou (Burkina Faso), Kisumu (Kenya) and Dar es Salaam (Tanzania). By integrating health promotion with environmental sustainability, the study will target key behavioural and environmental drivers, including poor diet, physical inactivity and school-level environmental conditions. The aim is to create an ecosystem where positive health behaviours are encouraged in tandem with sustainable environmental practices, such as school gardens and waste management.

Using co-design and school-based cluster-randomised controlled trials (cRCTs), Changemaker will evaluate the effectiveness and cost-effectiveness of a multi-component intervention combining sustainable health education, school gardening and waste management, motivational strategies and mass media campaigns. The study will simultaneously assess implementation outcomes such as feasibility, acceptability, appropriateness and adoption across diverse cultural settings. This type 2 hybrid effectiveness-implementation approach provides a comprehensive understanding of what works, for whom and under what conditions, supporting the development of scalable and sustainable solutions.^9^

## Methods and analysis

### Patient and public involvement

The trial, grounded in human-centred design, is based on a four-stage iterative co-design process using interviews, focus groups, photo-elicitation, workshops and evidence-based intervention development. Adolescents, parents, teachers and multisectoral stakeholders were engaged from the outset and throughout all stages of the study, ensuring continuous involvement and fostering their agency and active participation.

### Hypothesis and objectives

This hybrid type 2 implementation-effectiveness trial is guided by two hypotheses:

Hypothesis 1: The implementation of the multi-component sustainable health intervention over one school year will improve the primary effectiveness outcomes among participating adolescents. The primary effectiveness outcomes are mid-upper arm circumference z-score (MUACz) and hemoglobin concentration (Hb).

Hypothesis 2: Improvements in capabilities (knowledge and practices), opportunities (participation, fidelity and adoption) and motivation (acceptability, feasibility and appropriateness) among participating adolescents will lead to these measurable improvements in effectiveness outcomes.^18^

The objectives of this study are twofold:

Effectiveness objectives: To assess the effectiveness of the sustainable health intervention on health, nutrition and environmental outcomes among adolescents in three rapidly urbanising cities in Burkina Faso, Kenya and Tanzania. The study will evaluate whether the Changemaker intervention improves the primary outcomes of MUACz and Hb among adolescents. Furthermore, the effectiveness objective encompasses determining the effectiveness on secondary outcomes, including diet quality, physical activity, water, sanitation and hygiene (WASH) knowledge, mental well-being and household food insecurity.

Implementation objectives: To evaluate the processes and cost-benefits of the sustainable health intervention through the feasibility and potential utility of the contextualised implementation. This objective encompasses understanding the implementation processes of the Changemaker intervention, including how community-specific contextualisation shapes its delivery, adaptation and sustainability of the intervention through evaluating fidelity, dose, reach and quality of delivery for each intervention component. Furthermore, the implementation objective is to assess the acceptability, appropriateness, feasibility and adoption of the intervention across diverse study settings.

The effectiveness assessment will be guided by the primary research question: ‘What are the magnitudes of the intervention effects on nutrition, health and environmental outcomes after a one-school-year intervention implementation’. The primary effectiveness outcomes are (1) MUACz as a global marker of nutrition and (2) Hb as a global marker of human health. A 1-year follow-up is expected to be adequate for detecting intervention-related changes in Hb, as previous nutrition education and dietary intervention studies have reported significant improvements in Hb within approximately 3–6 months.^19 20^ Secondary effectiveness outcomes include knowledge level about waste management as a marker of environmental health, nutrition literacy and nutrition fluency, WASH knowledge, school gardening knowledge, mental well-being and food insecurity.

The implementation assessment will be conducted through an implementation evaluation in the study. This evaluation is guided by three key research questions: (1) How are the Changemaker intervention components and implementation strategies adapted and delivered across different country contexts, and with what level of fidelity (ie, adherence, dose, quality, participant responsiveness and programme differentiation*)*? (2) What are the key implementation outcomes as assessed through this evaluation (ie, acceptability, appropriateness, feasibility, adoption, reach and fidelity) and how do they influence the delivery of the intervention? (3) To what extent does the Changemaker intervention lead to changes in adolescent health behaviours, through which mechanisms, and for whom does it work under what contextual conditions? The process evaluation questions will be addressed using mixed methods to generate actionable insights on both implementation process and effectiveness required for scaling and sustainability. The primary implementation outcomes include participation and emotional and cognitive engagement. The secondary outcomes include acceptability, adoption, feasibility, fidelity, implementation cost and sustainability.

### Trial design

The study will comprise three independent, parallel-group,^21^ cRCTs across three study sites. Each trial will follow a hybrid type 2 approach, integrating effectiveness, process and economic evaluations. The intervention will follow harmonised intervention protocols, with context-specific adaptations. A hybrid type 2 approach allows for the simultaneous evaluation of intervention effectiveness and implementation processes, providing both rigorous outcome assessments and insights into real-world applicability. Trial clusters (i.e., schools) will be randomly allocated in a 1:1 ratio to the intervention and control arms. Intervention outcomes will be measured at baseline (month 1) and endline (end of school year). The active intervention period is planned for one school year between September 2025 and December 2026. As the intervention is behavioural in nature, blinding of participants, investigators and study personnel will not be possible.

### Participants, interventions and outcomes

#### Study setting

Changemaker will take place in Ouagadougou (Burkina Faso), Kisumu (Kenya), and Dar es Salaam (Tanzania). Table 1 presents key characteristics of the study areas, highlighting their unique geo-economic and sociodemographic features.^22–24^

**Table 1:** Characteristics of the study populations in Ouagadougou (Burkina Faso), Kisumu (Kenya) and Dar es Salaam (Tanzania)^22–24^

Characteristics	Ouagadougou, Burkina Faso	Dar es Salaam, Tanzania	Kisumu, Kenya
National GDP (2022)	US$893	US$1099	US$2082
Healthcare facilities	170 health facilities: one national hospital, two university hospitals	536 health facilities: 4 national hospitals, 38 public hospitals, 58 health centres, 436 public dispensaries	62 health facilities: 3 public hospitals, 6 public health centres, 21 public dispensaries
Geographic position	West Africa, Central in the country, landlocked	East Africa, North-East in the country, located at the coast of Indian Ocean	East Africa, South-West in the country, located by Lake Victoria
Area	520 km^2^	1493 km^2^	297 km^2^
Altitude	305 m above sea level	55 m above sea level	1131 m above sea level
Population size	3.6 million	5.4 million	404 160
Start of Health and Demographic Surveillance System (HDSS)	Ouagadougou 2008	Dar es Salaam 2011	Manyatta-Kisumu 2017
Number of secondary schools	120 public and 30 private schools	138 public and 186 private schools	112 public and 44 private schools
Weather/climate	Rainy season: June to October Mean annual rainfall: 800 mm Daily min T: 20°C–28°C Daily max T: 29°C–37°C	Rainy season: April to June Mean annual rainfall: 1114 mm Daily min T: 22°C–25°C Daily max T: 27°C–32°C	Long rains: March to May Short rains: October to December Mean annual rainfall: 725–1200 mm T between 17°C and 35°C
Major crops in subsistence agriculture	Millet, sorghum, maize, peanuts, rice and sesame	Maize, wheat, rice, sweet potatoes, bananas, beans, sorghum and sugar cane	Maize, beans, sweet potatoes, sorghum, rice and cassava
Adolescents’ nutrition status	18% BMI≥25 kg/m^2^ 15% anaemia	7% BMI≥25 kg/m^2^ 30% anaemia	20% BMI≥25 kg/m^2^ 20% anaemia
Average fruit and vegetable intake	146 g/day	167 g/day	192 g/day
Insufficient physical activity in adolescents (%)	79	82	87

BMI, body mass index; HDSS, Health and Demographic Surveillance System.

### Eligibility criteria

All schools in the study must be public or private institution in the urban setting of Ouagadougou, Kisumu and Dar es Salaam, either part of or in close proximity to a Health and Demographic Surveillance System (HDSS). Eligible schools must meet the following criteria: no existing school garden, access to year-round water, land suitable for establishing a garden and sufficient adolescent enrolment to accommodate the study sample.

The trial participants (adolescents) will be recruited using consistent criteria across all three sites. The inclusion criteria are: residence in the urban catchment area of Ouagadougou, Kisumu or Dar es Salaam; age between 12 and 16 years; enrolment in a selected secondary school within the catchment area; written informed consent provided by guardians (parents and legal guardians); and written informed assent provided by the adolescents.

Exclusion criteria include: Once a school is selected, adolescents within the specified school and grade will be excluded from participation if they do not meet the trial inclusion criteria. Additionally, adolescents with chronic illnesses, severe physical disability or who are pregnant will be excluded from participation.

### Consent and assent

Informed written consent will be obtained by data collectors at each study site. In accordance with international and national ethical guidelines on conducting research among minors, adolescents (<18 years) will be asked to provide informed assent as well as informed consent from a parent or guardian. Adolescents will be given time to reflect and ask questions before deciding to participate. Information about the study and the use of collected data will be provided both orally and in writing in national and relevant local languages, using materials that are accessible and understandable to adolescents and their guardians. Adolescents will have the right to decline participation even if their guardians provide consent and may withdraw from the study at any time.

All guardians, teachers and adolescents in the study will provide written informed consent and assent, respectively, for use of their data. Consent and assent forms can be found in online supplemental materials 2 and 3, respectively. All participants recorded, photographed and/or videotaped will provide written informed consent for the use of their recorded material on the Changemaker website, in printed material and during presentations.

### Additional consent provisions for collection and use of participant data and biological specimens

This trial is fully non-invasive and thus does not involve collecting or storing biological specimens.

### Interventions

#### Explanation for the choice of comparators

In Ouagadougou, Kisumu and Dar es Salaam, we will screen secondary schools in the catchment area or vicinity of the HDSS sites. In each country, we will enrol adolescents from four different schools. At each study site, two schools will serve as controls and two schools will receive the full intervention. The allocation to the intervention or control arm will be done at random. As the ‘standard of care’ package, the control arm schools will be exposed to the mass media campaign component guided by the WHO Best Buys on healthy lifestyles.^25^ This approach was adopted for ethical reasons, as mass media interventions are recommended as population-level strategies for promoting healthy diets and physical activity. The intervention effect will therefore be evaluated based on the added value of the school-based components delivered exclusively in intervention schools.

### Intervention description

#### Changemaker intervention

The Changemaker intervention comprises four components selected through a literature review and prior experience across diverse settings: (1) school gardening and organic waste composting^11 26–28^; (2) classroom-based sustainable health education^28–31^; (3) health talks using motivational strategies^32–34^; and (4) mass media campaigns promoting healthy behaviours.^35–37^ These interventions were chosen for their strong evidence in improving nutritional status and fostering healthy habits through individual behaviour change and structural shifts. Using a whole-systems approach, Changemaker operates across multiple social-ecological levels (ie, with adolescents, peers, parents, teachers, school system and communities) to co-design some aspects of the intervention components and their implementation, addressing dietary and physical activity behaviours and broader NCD risk factors. The intervention aligns with the COM-B model (Capability, Opportunity, Motivation-Behaviour), a framework recognising that behaviour change occurs through strengthening skills and knowledge (Capability), improving environmental and social conditions (Opportunity) and reinforcing psychological and emotional drivers (Motivation).^38^ Specifically, the intervention will build adolescents’ capability through school gardening and motivational strategy-led health education, enhance opportunity by creating supportive environments through mass media campaigns and school initiatives (school gardening, waste management and composting and classroom-based sustainable health education) and strengthen motivation by using motivational techniques to align health behaviours with adolescents’ context, values and goals. In this process, “Changemakers” (i.e., adolescents, teachers, parents, health workers and community members) play an integral role by acting as catalysts and leaders in the promotion of healthy behaviours, fostering peer engagement and creating a supportive culture for change within schools and communities.

The individual intervention components in Changemaker have been shown to be safe and efficacious in other settings.^39^ The mechanisms of change for each component are illustrated in table 2.

**Table 2:** Mechanisms of change for each intervention component

Intervention components	Effectiveness (evidence, including mechanism of change)
(1) School gardening and organic waste composting	Mechanism of change for reducing unhealthy diet risk factors: Increase adolescents’ fruits and vegetables intake by eating produce from school and urban farms or gardens^11 22^ Increase families’ fruits and vegetables intake through creation of school gardening at home and if produce brought home from school^26^ Improve adolescents’ willingness to try different fruits and vegetables by helping in school gardening—increases intake later in life^44 55^ Mechanism of change for reducing physical inactivity as a risk factor: Involving adolescents in the making of, tending to and harvesting from the school gardens (including making the compost) increases their time outside and physical activity^27 56^
(2) Sustainable health modules for classrooms	Nutrition education and physical activity promotion in the classroom leads to an increase in fruits and vegetables intake and reduction in physical inactivity^31^ Knowledge about (and trying) new foods increases the willingness to try new foods^30 44^
(3) Health talks using motivational strategy (MS) techniques	Health talks using MS leads to an increase of 1.3 servings per day of fruits and vegetables^57^
(4) WHO Best Buys: mass media campaign	Mass media campaigns targeting fruit and vegetable consumption can increase the consumption of fruits and vegetables by an average of 0.28 servings per day^35 36^ Increases nutrition knowledge and fruits and vegetables intake^58^

Some aspects of the four components will be co-designed by multi-sectoral stakeholders, including students, parents, teachers, school principals, representatives of the ministries of education, agriculture and health, as well as community representatives (e.g., nurses, community health workers, agricultural extension workers and informal community leaders). The delivery mechanisms will be contextualised. The details of the above will be published in a separate co-design methodological paper.

The intervention will be delivered over the course of one school year. School gardening and composting activities will be introduced at the beginning of the school year and sustained continuously. Sustainable health education modules will be added into the existing school curriculum when possible or conducted in extra-curricular time and delivered by trained schoolteachers using participatory methods. Health talks employing motivational strategies will be conducted by trained staff to engage adolescents in personalised goal setting and behaviour change support. Mass media campaigns will be disseminated throughout the intervention period to reinforce key healthy behaviour messages. Delivery agents will include teachers, health workers, agricultural specialists and others. The delivery agents will be trained and supported through a capacity-building programme to ensure consistent and effective delivery of the intervention across all sites.

The control arm will receive the standard school-based health education programme, reflecting the usual practices in schools, along with the mass media campaigns and serve as a comparison to measure the additional impact of the Changemaker intervention.

### Implementation strategies for intervention components

The multi-component Changemaker intervention is grounded in established implementation and behaviour-change theory, drawing on the Expert Recommendations for Implementing Change framework,^40^ the COM-B model for Behaviour Change,^18^ the Normalisation Process Theory (NPT)^41^ and the Practical, Robust Implementation and Sustainability Model (PRISM).^42^ In addition to this theoretical base, the intervention employs tailored implementation strategies informed by country-specific context assessment and co-design workshops.

These implementation strategies aim to strengthen participation and engagement (primary implementation outcomes) as well as acceptability, adoption, feasibility, fidelity, implementation cost and reach sustainability (secondary implementation outcomes) across school and community settings. These strategies include: (1) build synergies between intervention components; (2) align with national and local policies; (3) adapt to local context and existing structures; (4) develop user-friendly delivery manuals and materials; (5) develop flexible delivery methods; (6) train delivery agents; (7) build community engagement and develop partnerships; (8) secure and mobilise resources; (9) use monitoring and implementation support.

For school gardening and organic waste composting, implementation focuses on embedding these practices into the school environment through alignment with national and local policies, fostering legitimacy and institutional buy-in. The strategy includes developing user-friendly manuals, offering flexible delivery options (e.g., vertical or container gardening) and co-designing activities with students and teachers to fit local conditions and contexts. Training and regular support from agricultural extension workers, collaboration with local organisations and resource mobilisation efforts will promote sustainability and adoption. These activities are reinforced through other components, such as the sustainable health modules and mass media campaigns, creating a synergistic effect on behaviour change and environmental awareness.

For the sustainable health module, implementation strategies emphasise curriculum integration when possible. The health modules are aligned with educational standards and designed for adaptability, using participatory and experiential learning methods that resonate with adolescents. Training and capacity building for teachers (i.e., delivery agents) ensure fidelity of delivery, while user-friendly materials and flexible schedules support feasibility. Co-design with educators and students will enhance positive implementation outcomes, and community engagement efforts will help extend health promotion beyond classrooms. Feedback mechanisms and iterative adjustments ensure the modules evolve with the needs and contexts of schools.

For health talks using motivational strategies, implementation involves training designated facilitators in adolescent-centred communication techniques. These sessions are structured to foster personal goal setting, address barriers and reinforce behaviour change through tailored support. Alignment with the sustainable health modules and other activities helps normalise conversations around nutrition, physical activity and well-being. Peer-to-peer encouragement and trust-building are emphasised as key mechanisms for engagement.

For the mass media campaigns promoting healthy habits, implementation strategies include flexible delivery methods using multi-platform dissemination through school announcements, social media, posters and community radio. Messaging is designed to complement and amplify the other components, promoting consistency, reinforcement and synergy across settings. Community media personnel and youth advocates are trained to ensure relevance and relatability of content. Ongoing monitoring of message reach and resonance enables iterative refinement and responsive communication.

Together, these implementation strategies support the preparation, delivery and monitoring of a cohesive, context-responsive intervention that is designed to be scalable, sustainable and impactful across multiple levels of the social-ecological system.

### Methods and data collection for process evaluation

The Changemaker project uses a hybrid type 2 implementation-effectiveness cRCT approach, which allows for simultaneous evaluation of the intervention’s impact on health outcomes and the processes critical to its successful execution. This dual-focus approach is anchored in the two key implementation science frameworks mentioned previously PRISM and NPT. PRISM ensures that contextual factors, such as organisational readiness, stakeholder dynamics and external policy environments are systematically assessed, while NPT provides a lens to evaluate how the intervention becomes embedded in routine practice through coherence (i.e., sense-making and mutual win-win), cognitive participation (ownership and advocacy), collective action (roles, responsibilities and resources) and reflexive monitoring (learning and improving). The mixed-methods evaluation captures both quantitative and qualitative dimensions of implementation.

Quantitative fidelity metrics, including adherence (delivery as planned), dosage (exposure levels) and quality (competence in delivery) will be tracked using structured logs by school-based implementation teams, periodic intervention audits by country-specific Changemaker implementation teams, and standardised feedback loops. For example, adherence to school gardening sessions will be documented through setup checklists and attendance records, while dosage will be measured by student participation rates across all intervention components. To provide a comprehensive assessment, the study integrates multiple data sources. Fidelity is quantified using a Composite Fidelity Score (CFS), which aggregates weighted scores for adherence, dosage and quality across the four core components (school gardening, health education, health talks and mass media).

Qualitative insights will be derived from participatory workshops with a body of stakeholders at each intervention school, who include students, teachers and community leaders. These workshops will employ innovative tools like ripple effects mapping to visualise unintended consequences (e.g., community interest in school gardening) and user journey mapping to identify barriers and facilitators at different stages of engagement. By conducting these workshops at mid-intervention and postintervention implementation, the evaluation will ensure real-time adaptation and foster local ownership, enhancing the intervention’s cultural relevance and sustainability. In-depth interviews with teachers and focus groups with students will reveal challenges such as time constraints or resource limitations, while also highlighting successful strategies like peer-led gardening activities. Cross-country comparisons will further illuminate how sociocultural and infrastructural differences, such as varying levels of urban density or food insecurity, shape implementation, while preserving its core principles, ultimately maximising its potential for long-term impact on adolescent health.

### Plans for assessment and collection of outcomes

The quantitative and qualitative data will be collected in person by trained research assistants. The questionnaire-based data and data from physical examinations will be collected on Android-operating smartphones employing the Open Data Kit platform (ODK, Get ODK, USA). This allows customised data collection tools and forms that are created and processed to generate .csv files. In addition, for Hb measurements, we employ the EzeCheck device which uses spectrometry to estimate Hb.^43^ As indicated above, the outcomes of the process evaluation will be measured through workshops, self-reported quantitative data, and monitoring activities. Also, qualitative data from participants and implementers of the intervention will be collected through in-depth interviews, semi-structured interviews and focus group discussions. Table 3 shows the assessment tools for the intervention effectiveness outcomes.

**Table 3:** Trial outcome indicators, outcome measures and assessment tools for the intervention effects

	Outcome measure	Assessment tool
Primary effectiveness outcomes		
Nutrition marker	Mid-upper arm circumference z-score (MUACz)	MUAC z-score tape
Health marker	Haemoglobin concentration (Hb)	EzeCheck device
Secondary effectiveness outcomes		
Nutrition and health indicators		
Nutritional status	Body weight, height, body mass index (BMI)	Weighing scale, stadiometer (both devices SECA, Germany)
Diet quality	A priori indices of diet quality, such as Global Diet Quality Score, Women Dietary Diversity Score, Planetary Health Diet Index, including alcohol consumption	Culturally adapted Food Propensity Questionnaire (FPQ)
Nutrition literacy and fluency	Achieved scoring in nutrition literacy and fluency	22 questions on nutrition literacy and fluency
Food Insecurity	Food Insecurity Experience Scale Survey (FIES-SM) 30 days	Eight questions on individual experience over the last 30 days
Physical activity	Categories of moderate physical activity at 3–6 metabolic equivalents of task (METs). Vigorous physical activity at 6 METs	Two questions on physical activity per week and on a typical day
Water, sanitation and hygiene (WASH) measures	Achieved scoring in knowledge on WASH measures	Two questions on handwashing
Mental health	Psychological well-being and quality of life, including anxiety and depression	The Warwick-Edinburgh Mental Well-being Scale (WEMWBS); Short-Form Survey Instrument (SF-36)
Environmental indicators		
Waste management—knowledge and practice	Achieved scoring in knowledge; number of dustbins in schools and communities	Three questions on waste disposal (WHO/UNICEF 2018)
Knowledge of school gardening practice	Achieved scoring in knowledge	21 questions Changemaker tool
Economic indicators		
Socioeconomic status of the household	International Wealth Index (IWI)^59^	Health and Demographic Surveillance (HDSS) questionnaire on assets and consumer durables

Each data collector will use a password-protected device for data collection. During the data collection periods, raw data files, including audio transcripts of workshops, interviews and group discussions, will be stored on password-protected computers, in an encrypted format. All data will be deleted from smartphones and audio recorders as soon as they are safely stored on the central server at the University of Bonn (UoB) Computing Center.

### Plans to promote participant retention and complete follow-up

To promote participant retention and ensure complete follow-up, we will maintain regular engagement with participants through scheduled activities and consistent communication. Additionally, collaboration with school staff and other local stakeholders will help reinforce the importance of continued participation and follow-up assessments.

### Criteria for discontinuing or modifying allocated interventions

If natural or human-made disasters occur, including pandemics, epidemics, armed conflict or ethnic violence, data collection may be suspended in part or in full. Individual criteria for discontinuation are: the participant or guardian withdraw their consent/assent; the participant requests to discontinue the interview or the physical examination, the participant is found not to meet the original inclusion criteria; or the participant is untraceable. The study participants who withdraw can choose to have their data destroyed at the time of withdrawal. For these withdrawn participants, we will only keep an absolute count of the number of withdrawals with no linkable information to the individual. If withdrawn participants do not request to have their data destroyed, we will maintain and use their data for analysis and publication.

### Strategies to improve adherence to interventions

Many of the study implementation strategies are designed to improve intervention adherence. Adherence will be supported by regular contacts with beneficiaries during training, workshops and data collection. We will closely monitor attendance and participation throughout to keep participants motivated and engaged. The project team will also closely collaborate with the local stakeholders, the participating schools and ministries of health, agriculture and education. In addition, fidelity will be closely monitored as part of the process evaluation, referring to the extent to which the intervention is^44^ delivered as intended, encompassing adherence; integrity; and quality of delivery.^31^

### Relevant concomitant care permitted or prohibited during the trial

We anticipate minimal to moderate risks associated with participation in the trial. The intervention and data collection methods will be non-invasive. When participants are identified as under- or over-nourished (determined by MUACz and body mass index (BMI)), their guardians will be informed of the measurements and provided with an explanation of the interpretation of these values. When a participant is found to have anaemia (measured by EzeCheck device and App, EzeRx Health Tech, India), the participant and guardians will be informed of the anaemia status, which indicates the participant’s Hb and the WHO cut-offs. Participants and guardians will be informed of any abnormal findings, such as low Hb levels, and advised to seek follow-up care at a local health facility. Similarly, if concerns related to mental health or sexual and reproductive health arise during data collection, adolescents and their guardians will be guided towards appropriate information and care pathways. All routine preventive and curative healthcare services remain accessible to participants throughout the trial.

### Provisions for post-trial care

Participants may find some items in the questionnaire modules to be personal, and thus, may experience some discomfort. They will be advised that they can choose not to respond to any questions that make them feel uncomfortable, without any ramifications. Power imbalance may occur when adolescents interact with older participants, teachers and health personnel. To address this, our project will set out ground rules on how to collaborate and how to solve conflicts or issues. Additionally, all researchers involved in the study will be required to adhere to established principles of respectful engagement with adolescents to minimise any risk of physical, verbal or emotional harm. Research staff will receive training on recognising and appropriately managing power imbalances throughout the research process.

If the research staff identifies information that requires professional intervention (e.g., abuse or health problems), we will refer the adolescent to professional services for help and support. For instance, when participants are found to have anaemia, measured by EzeCheck, the participant and parents will be informed and receive a referral slip for local health facilities, which indicates the participant’s Hb and WHO cut-offs. If a participant’s measurements (MUACz and BMI) indicate that they may be above or below the healthy range, they and the family will be informed of their results and provided with an explanation of what the values mean. Clear ground rules that highlight respect for peers will be explained and highlighted during information meetings, the recruitment process and the data collection process. No provision of post-trial care will be made.

### Outcomes

This trial has primary and secondary effectiveness outcomes for intervention effects. We consider one nutrition-related primary outcome, MUACz, and one health-related primary outcome, Hb. Table 3 shows the primary and secondary effectiveness outcomes for the intervention effects.

The process evaluation will assess both primary and secondary outcomes linked to key impact pathways, including activities, inputs and outputs. This will be achieved through comprehensive mixed-method data collection and analysis. Qualitative data collection will be complemented by quantitative data, embedded in the questionnaire for outcome assessments with participants, and ongoing monitoring (logs and observations) to track implementation fidelity. This mixed-methods approach will offer a comprehensive understanding of how the intervention processes contribute to desired outcomes outlined in table 4.

**Table 4:** Outcomes and indicators of the process and cost evaluations

Outcome indicator	Outcome measure	Data collection source
Primary implementation outcomes		
Participation	Completion rates against target Proportion of intervention sessions attended Student attendance	Fidelity assessment (including logbooks and audits), during and after intervention implementation evaluation using focus group discussions and semi-structured interviews
Emotional and cognitive engagement	Emotional connection (interest and motivation) and cognitive involvement in intervention activities (understanding and reflection on content)	During and after intervention implementation evaluation using focus group discussions and semi-structured interviews
Secondary implementation outcomes		
Acceptability	Physical, logistical and socioeconomic accessibility and inclusivity of intervention activities for all students, teachers and other school community members Perceived effort required to participate or conduct the intervention Alignment of the intervention (content and delivery methods) with participant’s values and cultural norms Confidence in understanding or implementing the intervention Gender-sensitivity of intervention components and materials Satisfaction with various aspects of the intervention (e.g., content, complexity, comfort, delivery and credibility)	Fidelity assessment, monitoring reports, during and after intervention implementation evaluation using focus group discussions and semi-structured interviews
Adoption	Uptake, utilisation, and intention to try among stakeholders and through observations of the initial implementation Support and school leadership and educational authorities to implement and sustain the intervention activities Alignment of intervention to be compatible with the school’s culture, context, priorities and existing policy frameworks/guidelines	Fidelity assessment, monitoring reports, during and after intervention implementation evaluation using focus group discussions and semi-structured interviews
Feasibility	Actual fit or utility of the intervention with school schedules, resources and routines Availability and adequacy of necessary infrastructure, materials and staff training Ability to deliver the intervention as designed while accommodating contextual variations Suitability of the intervention for everyday use Practicability of delivering the intervention components as designed	Fidelity assessment, monitoring reports, during and after intervention implementation evaluation using focus group discussions and semi-structured interviews
Fidelity	Delivered as intended Adherence Integrity Quality of programme delivery	Fidelity assessment, monitoring reports, during and after intervention implementation evaluation using focus group discussions and semi-structured interviews
Implementation cost	Implementation cost	Fidelity assessment, monitoring reports, semi-structured interviews
Reach	Penetration Demographic representation Level of institutionalisation Services access Equity in access	Fidelity assessment, monitoring reports, during and after intervention implementation evaluation using focus group discussions and semi-structured interviews
Sustainability	Maintenance Continuation Durability Incorporation and integration Institutionalisation Sustained use Routinisation	During and after intervention implementation evaluation using focus group discussions and semi-structured interviews

### Participant timeline

The Changemaker project officially kicked off in February 2024. Baseline assessments are estimated to be conducted in Kenya in September 2025, in Burkina Faso in November 2025 and in Tanzania in January 2026. The starting date of the baseline and implementation will be based on a multitude of criteria, including the school calendar and the school administration preferences. Baseline and endline data collection schedules are aligned with the academic calendars of each participating country. Although assessments will take place at different calendar times across countries, data collection within each country will occur during comparable school-year periods. Statistical models will further adjust for country- and time-specific effects to account for potential seasonality and contextual differences. At each site, intervention participants will be followed up for one school year after baseline assessment and the start of the intervention (figure 1 and table 5). Participants in the control arm schools will be exposed to the mass media campaign. We will monitor inputs, processes and outputs throughout the intervention phase. Finally, we will conduct endline assessments for the primary and secondary effectiveness outcomes. The anticipated end of data collection is January 2027, and the study ends in December 2027 (see online supplemental file 1).

**Figure 1:**
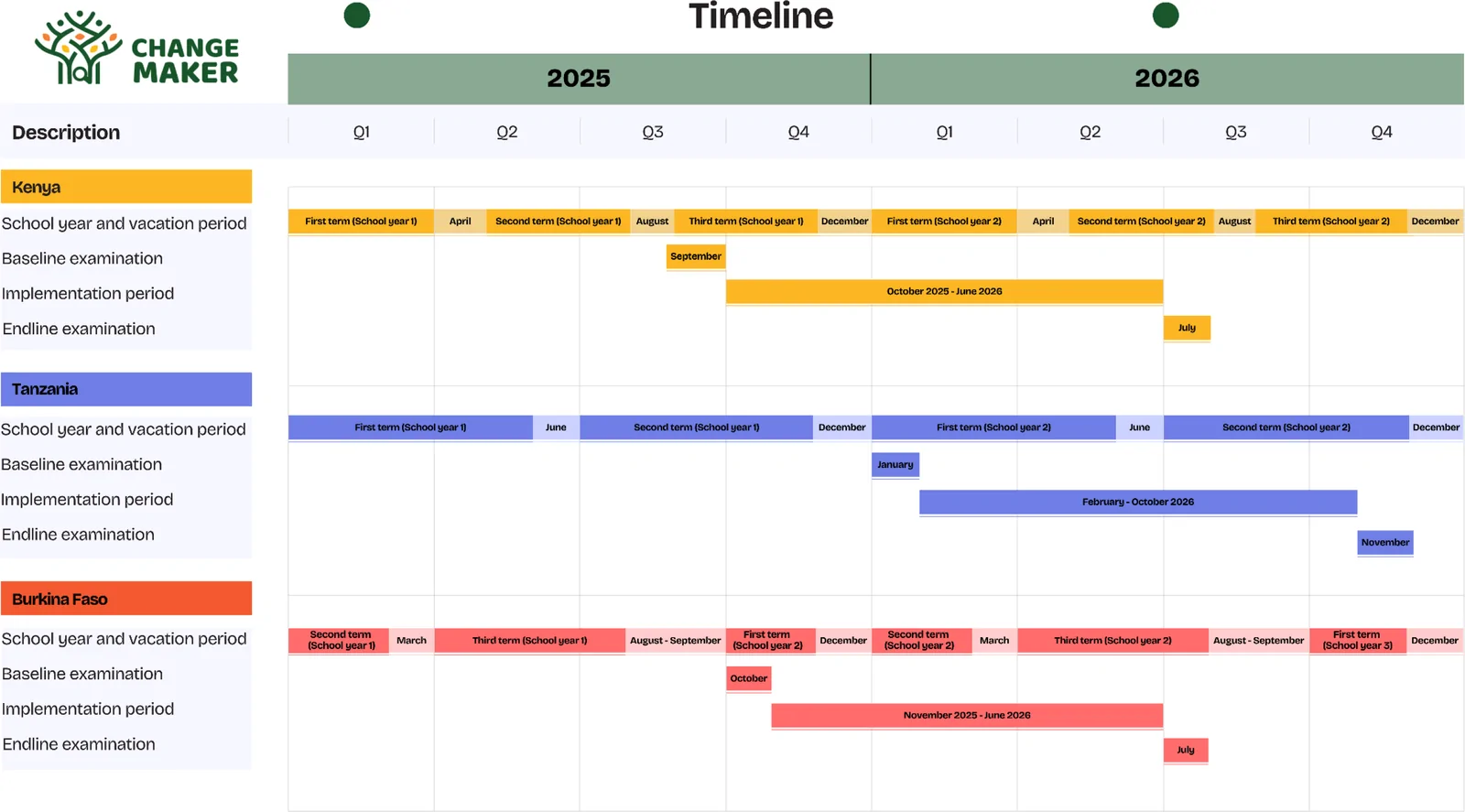
The implementation timeline in Kenya, Burkina Faso and Tanzania.

**Table 5:** Schedule of enrollment, interventions and assessments (Standard Protocol Items: Recommendations for Interventional Trials (SPIRIT) figure)

	Trial period					
	Timepoint	Enrollment	Intervention implementation			Close-out
		Pre-academic year	Academic year			Post-academic year
			Trimester 1	Trimester 2	Trimester 3	
	**Enrollment**					
	Eligibility screening	X				
	Informed consent	X				
	Informed assent	X				
	Randomisation	X				
	**Intervention/control**					
Intervention	Sustainable health education		X	X	X	
	School gardening		X	X	X	
	Waste management		X	X	X	
	Motivational strategies		X	X	X	
	Mass media campaigns		X	X	X	
Control	Mass media campaigns		X	X	X	
	**Assessments: primary effectiveness outcomes**					
Nutrition marker	Mid-upper arm circumference z-score		X		X	
Health marker	Haemoglobin concentration		X		X	
	**Assessments: secondary effectiveness outcomes**					
Nutritional status	Body weight, height, body mass index (BMI)		X		X	
Diet quality	A priori indices of diet quality, such as Global Diet Quality Score, Women Dietary Diversity Score, Planetary Health Diet Index, including alcohol consumption		X		X	
Nutrition literacy and fluency	Achieved scoring in nutrition literacy and fluency		X		X	
Food Insecurity	Food Insecurity Experience Scale Survey (FIES-SM)		X		X	
Physical activity	Categories of moderate physical activity at 3–6 metabolic equivalents of task (METs). Vigorous physical activity at 6 METs		X		X	
Water, sanitation and hygiene (WASH) measures	Achieved scoring in knowledge on WASH measures		X		X	
Mental health	Psychological well-being and quality of life, including anxiety and depression		X		X	
Waste management—knowledge and practice	Achieved scoring in knowledge; number of dustbins in schools and communities		X		X	
Knowledge of school gardening practice	Achieved scoring in knowledge		X		X	
Socioeconomic status of the household	International Wealth Index (IWI)		X		X	
	**Assessments: primary implementation outcomes**					
Participation	Completion rates against target Proportion of intervention sessions attended Student attendance		X	X	X	
Emotional and cognitive engagement	Emotional connection (interest and motivation) and cognitive involvement in intervention activities (understanding and reflection on content)		X	X	X	
	**Assessments: secondary implementation outcomes**					
Acceptability	Satisfaction with various aspects of the intervention (eg, content, complexity, comfort, delivery and credibility) Physical, logistical and socioeconomic accessibility and inclusivity of intervention activities for all students, teachers and other school community members Perceived effort required to participate or conduct the intervention Alignment of the intervention (content and delivery methods) with participants’ values and cultural norms Confidence in understanding or implementing the intervention Gender-sensitivity of intervention components and materials		X	X	X	
Adoption	Determine the uptake, utilisation, intention to try among stakeholders and through observations of the initial implementation Support and school leadership and educational authorities to implement and sustain the intervention activities Alignment of intervention to be compatible with the school’s culture, context, priorities and existing policy frameworks/guidelines		X	X	X	
Feasibility	Actual fit or utility of the intervention with school schedules, resources and routines Availability and adequacy of necessary infrastructure, materials and staff training Ability to deliver the intervention as designed while accommodating contextual variations Suitability of the intervention for everyday use Practicability of delivering the intervention components as designed		X	X	X	
Fidelity	Delivered as intended; adherence; integrity; quality of programme delivery		X	X	X	
Implementation cost	Implementation cost		X	X	X	
Reach	Penetration; demographic representation, level of institutionalisation; services access; and equity in access		X	X	X	
Sustainability	Maintenance; continuation; durability; incorporation; integration; institutionalisation; sustained use; routinisation		X	X	X	

### Sample size

Our sample size calculation was based on the primary effectiveness outcome of MUACz. The MUACz tape is a novel tool that has not been used in previous intervention studies.^45^ Therefore, we relied on unpublished piloting data of variations in MUACz in the target populations. The observed means ranged from –0.32 in girls to –1.06 in boys, each with an SD of 1. We applied the hypothesis test for two means in a cluster-randomised design using the PASS 2024 software (Power Analysis & Sample Size, NCSS Statistical Software, V.24.0.5, UT, USA).

For this trial, we will enrol adolescents in 12 different schools, representing six clusters per intervention arm. The intraclass correlation coefficient within schools was estimated at 0.025,^46^ representing moderate heterogeneity. Based on differences in mean MUACz observed during piloting and assuming an SD of 1, the sample size of 154 adolescents per school will enable us to detect between-group differences in mean MUACz of 0.3 or greater with statistical power (1−β) of 80% and an α-level of 0.05 (two-sided). To account for 20% loss to follow-up, we aimed to recruit 190 participants per school. These values are acceptable for trials designed to confirm, rather than establish, an intervention’s effectiveness.^47^ In addition to the members of the intervention task force, we will enrol 190 adolescents from each school, translating to 760 participants per country and a total sample size of 2280 participants.

### Recruitment

Participating schools will be selected based on the criteria described in the Eligibility criteria section. They will be recruited in close collaboration with partners who run the local HDSS cohorts in Ouagadougou, Kisumu and Dar es Salaam (table 1). These partners have excellent links with their respective communities and enjoy trusted relationships with key members to ensure successful selection of schools. The adolescent study participants will be recruited from the selected schools. For adolescents recruited at their school, information sharing about Changemaker will start in the classroom with the permission of teachers and principals. We will enrol 760 adolescents between 12 and 16 years from four different schools in each city (Ouagadougou, Kisumu and Dar es Salaam). Guardians and adolescents will receive written invitations for adolescents to participate through their teachers and community stakeholders. Guardians will be asked to consent first, thereafter adolescents will be asked for assent. If guardians do not consent, their adolescent will not be requested to assent, as they cannot participate. In cases when guardians consent, the adolescents will still be given the opportunity to assent or decline to participate.

### Assignment of interventions: allocation

#### Sequence generation and concealment mechanism

Randomisation will be conducted by researchers external to the engagement and selection of schools. This means that the randomisation will not be done by the same individuals who will conduct the examinations. Schools will be randomised into either the intervention group (two schools per site) or the control group (two schools per site). Randomisation will be conducted through simple randomisation at each study site. Schools will be randomised separately within each country using locally appropriate procedures. In Kenya, allocation will be conducted through a paper-draw method, whereby representatives from each school randomly select folded slips marked either ‘YES’ (intervention) or ‘NO’ (control). In Burkina Faso, schools will be randomised using Stata V.18. In Tanzania, schools will be assigned a random number generated in Microsoft Excel (RAND function), ranked accordingly and allocated to either the intervention or control arm based on the randomised order. We will minimise bias in the random allocation process to ensure the comparability of schools in both arms and maintain transparency.

#### Implementation

At each site, the main data officer will be responsible for allocation sequence, enrolment and assignment to the intervention.

### Assignment of interventions: blinding

#### Who will be blinded and procedure for unblinding

This behaviour change intervention will not involve blinding of participants or the implementation team; however, research assistants, data analysts and evaluation teams will remain blinded to the group allocation. Although the study design is openly labelled, any unblinding of results will occur only after blinded findings have been reviewed and interpreted by the Advisory Committee.

#### Data management

Changemaker has one central data manager based at UoB. This primary data manager is responsible for overseeing all data storage, management and use. Each study site also has one designated focal data manager and one institution’s data manager. During monthly meetings, local data managers will report their observations from plausibility checks and interim analysis to the primary central data manager and the steering committee.

##### Storage and processing

The Changemaker study team owns the research data collected in all phases of the project and has complete control over it. The data will be downloaded and safely stored for analysis on a computing system maintained by UoB in Germany. Within the study, we will collect personal information (such as participant name, date of birth, email address). All the personal identification data will be stored in password-protected files at the respective study sites, and only local study teams will have access to this data through the local data manager. All the personal data available on the hard copies will be stored in locked cupboards, and only local study teams will have access to this data through the local data manager. After de-identification by the local study teams, only de-identified (pseudonymised) data will be hosted on the UoB server. The collected data, which will not include any information that could be used to identify participants, will be analysed by the research team, and the results will be published. Only quantitative data will be made available to the scientific community following rules for open, transparent and reproducible research. Qualitative data will not be made publicly available, because this will violate the de-identification and thus confidentiality of participants’ data. As defined in the guidelines for good scientific practice, primary data will be stored and kept for a minimum of 10 years.

##### Sharing

At the end of the project, research data will be archived in line with the FAIR principles (findability, accessibility, interoperability, reusability). Where ethical approvals permit, anonymised data will be made openly available for other researchers and policymakers. All research reports and academic publications will be made open access to ensure anyone can access them without financial costs.

##### Data security

In this multi-centre study, each local institution will be responsible for data collection and management at their site. The study requires repeated contacts and visits with participants. Therefore, data collected by local study teams will be identifiable by phone numbers and names. However, unique participant identifiers (IDs) will be used to link participant information collected with anthropometric data, Hb and questionnaire data, as well as qualitative data (audio files, transcribed and translated interviews, focus group discussions and process documents). The key linking the IDs to the participants’ identifiers will be stored in a separate password-protected file, which will only be accessible to individuals given authorisation. The identifier key will only be available to the local study teams, and the broader international collaborators’ team will only be provided with the pseudonymised data.

Participants’ data will be entered into password-protected tablets and uploaded to a secure server at each study site. The linkage key to connect participants’ data with their identifiers will be stored separately. The data received by the international collaborators’ team will be pseudonymised with no phone numbers or names and stored on a secure platform using two-step authentication access, on password-protected, encrypted computers.

### Confidentiality

We ensure complete confidentiality of the data by following and conforming with the requirements and regulations of the German National Data Protection Regulation (Datenschutz-Grundverordnung – DSGVO). Data will be available exclusively to members of the study team instructed for complete confidentiality. Informed consent/assent forms, research data and other participant-related documents will be safely stored during the study at the HDSS research centres in Burkina Faso, Kenya and Tanzania. All data will be pseudonymised for analysis. The data will be stored securely as password-protected files on a central server with a two-factor identification system at the UoB Computing Center. Third parties will not have access to personal data.

### Plans for collection, laboratory evaluation and storage of biological specimens for genetic or molecular analysis in this trial/future use

Not applicable: Changemaker is designed to use non-invasive technologies.

### Statistical methods

#### Analysis of the primary and secondary outcomes

Based on an approved analysis plan, the intervention effects will be calculated for the total study population and by site. The data will be analysed as intention-to-treat and per protocol. For each study site, the baseline data will be presented by intervention arm per schools and per individuals. We will display means and SD for normally distributed continuous variables, and medians and IQRs for skewed data. Categorical variables will be presented as proportions (%) and counts.

For the intervention effects, we will calculate beta coefficients, 95% CIs and p values, using generalised linear mixed models (GLMM) to account for the cluster design by school. The GLMM can also be applied for binary outcomes. Pooled analysis across study sites will use meta-analysis with tests for heterogeneity and country-level fixed effects. No formal interim analysis will be conducted.

#### Methods for additional analyses (e.g., subgroup analyses)

The regression models for estimating the intervention effects will be adjusted for potential imbalances in baseline characteristics. In addition to adjustments for such imbalances, we will perform difference-in-differences (DID) analyses to control for unmeasured confounding. These DID estimates compare the change in outcome variables from baseline to endline examinations between the intervention arm and the control arm. Subgroup analyses may comprise stratification by gender, level of school class and size of the school, among others. Sensitivity analyses, exploratory analyses and moderator analyses may also be conducted based on post implementation data.

Intervention fidelity will be quantified through a CFS, with integrated weighted scores for adherence, dosage and quality across four components: school gardening, health education, motivational strategies and mass media. Qualitative data from stakeholder workshops, interviews and focus groups will be analysed using thematic analysis and grounded in the PRISM and NPT to explore contextual factors, adaptation processes and mechanisms of change.

Triangulation of data sources and methods will strengthen the validity of findings by cross-verifying patterns across quantitative scores, qualitative themes and contextual insights. Findings will be integrated through joint displays, aligning fidelity scores with qualitative narratives to explain patterns. Cross-country comparisons will further highlight how context influences outcomes. This participatory, theory-informed analysis will not only measure impact but also generate actionable insights for scaling and sustaining interventions in diverse LMIC settings.

#### Methods in analysis to handle protocol non-adherence and any statistical methods to handle missing data

We will conduct intention-to-treat analysis and compare the findings to per-protocol analysis. Multiple imputation will be considered for covariates only.

#### Plans to give access to the full protocol, participant-level data and statistical code

Data produced and published during the Changemaker project will be made openly accessible through Zenodo, a general purpose open-access repository developed under the European OpenAIRE programme and operated by CERN. All deposited material, including anonymised data, consortium-level data, metadata, analysis files and statistical code, will receive a DOI to make it easily accessible and citable. Anonymised consortium level data will be made openly available no later than 1 year after project completion. Users must acknowledge the Changemaker Consortium and cite the data source in any resulting publications. Quantitative data will be licensed under a Creative Commons Attribution 4.0 (CC BY 4.0), which permits redistribution, remixing, adaptation and commercial reuse with appropriate attribution.

### Oversight and monitoring

#### Composition of the coordinating centre and trial steering committee

Steering committee: Centre de Recherche en Santé de Nouna (Ali Sié), Muhimbili University of Health and Allied Sciences (Bruno Sunguya), Kenya Medical Research Institute-Kisumu (Erick M O Muok), University of Bonn (Ina Danquah), Karolinska Institutet Stockholm (Kristi Sidney Annerstedt, Helle Mölsted Alvesson), Joseph Ki-Zerbo University of Ouagadougou (Abdramane Soura), Africa Academy for Public Health (Mary Mwanyika Sando, Amani Kalokola Tinkasimile), Kisumu County Government—Department of Agriculture, Livestock and Fishery (Joseph Amoke), Technical University of Kenya (Alice Achieng Ojwang).

Coordination and management; evaluation of intervention effects: University of Bonn (Ina Danquah, Raissa Sorgho, Rhodah Nekesa, Nils Wortmann).

Coordination and management; process evaluation: Harvard T.H. Chan School of Public Health (Sachin Shinde, Wafaie Fawzi).

Coordination and management; health economics evaluation: Karolinska Institutet Stockholm (Kristi Sidney Annerstedt, Helle Mölsted Alvesson, Sibylle Herzig Van Wees, Claudia Hanson, Elin Larsson, Elisa Gobbo and Sofia Tzirita).

#### Composition of the data monitoring committee, its role and reporting structure

The project will operate with appointed data managers at each site and its steering committee, consisting of one representative of each participating institution. A formal data monitoring committee has not been established, because this trial constitutes a low-risk intervention. The intervention components are well-established routine interventions in many countries (education and gardening), and with low-risk populations. The research team will ensure strong data monitoring procedures, including a pre-approved data monitoring plan, regular review by the investigator’s team, institutional review board (IRB) review and regular safety checks. The project has one central data manager at the University of Bonn, who oversees all data storage, quality and use.

### Data monitoring: interim analysis

No formal interim analysis will be conducted considering the general safety of this early-stage nutrition intervention and the fact that this is a confirmatory trial.

### Adverse event reporting and harms

The implementation teams at each study site are responsible for monitoring, addressing and reporting adverse events. Possible adverse events include eco-anxiety, climate-related stress or grief, food insecurity-related anxiety, general food anxiety, body-image concerns and associated bullying. There are also possible adverse health risks such as food poisoning from contaminated soil and plants. In the event of any adverse outcome, the personal data of participants will be strictly kept confidential by the local teams. We will provide confidential reporting channels for participants to the proper authorities if they want to report the adverse events outside the study. IRBs will receive reports of serious adverse events. After the end of the study, a final report will be submitted to the IRBs with a summary of the results and conclusions of the study.

### Frequency and plans for auditing trial conduct

The project principal investigators, coordinators, data manager, site coordinators and data collection teams will meet online bi-weekly to review the conduct of the trial. During these meetings, the teams will review the adherence to intervention components, logistical aspects, feedback from school representatives and other important actors, completeness and quality of collected data, and adherence to milestones. In addition, we will create communication groups (short messages, phone calls) for regular exchange of trial progress.

### Plans for communicating important protocol amendments to relevant parties (eg, trial participants, ethical committees)

All modifications to the study protocol, which impact the study (trial design, implementation, evaluation), the participants (involvement, safety) or essential procedures and administrative aspects, will undergo a formal amendment to the protocol. Amendments will be agreed on by the steering committee and approved by all IRBs involved.

## Ethics and dissemination

The IRB of University Hospital and Medical Faculty, University of Bonn reviewed and approved the Changemaker study protocol and all its annexes in July 2024 (reference: 2024-265 BO). This serves as the principal approval from one of the coordinating institutions. In addition, the study protocol, its processes, and tools were reviewed and approved by local IRBs at the following institutions:

- Ethics committee approval from Bonn University in Germany—Reference number: 2024-265 BO (2024.11.21).
- Ethics committee approval from Karolinska Institutet in Sweden—Reference number: Dnr 2024-07452-01 (2024.12.03).
- Ethics committee approval from Institute National De Sante Publique (INSP) Committee, Burkina Faso Ouagadougou—Reference number: Dossier number A2024.09.12-08 (2024.11.04).
- Ethics committee approval from the national board in Burkina Faso—Reference number: Dossier number A2024.09.12-08 (2024.11.04).
- Ethics committee approval Kenya Medical Research Institute (KEMRI)—Scientific Ethics Review Unit (SERU), Kisumu and Nairobi, Kenya—Reference number: KEMRI/SERU/CGHR/01-09-517/5108 (2024.12.18).
- Ethics committee approval from National Board in Kenya—Reference number: Research License number NACOSTI/P/25/415277 (2025.01.13).
- Ethics committee Muhimbili University of Health and Allied Sciences (MUHAS), MUHAS Research and Ethics Committee, Dar es Salaam, Tanzania—Reference number: DA.282/298/01.C/2523 (2024.11.18).
- Ethics committee National Institute for Medical Research—National Health Research Ethics Committee (NatHREC), Dar es Salaam, Tanzania Reference number: NIMR/HQ/R.8a/Vol.IX/5008 (date 2025.06.26).

The study is registered on WHO’s International Clinical Trials Registry Platform (ICTRP) via the German Register for Clinical Trials (DRKS) under the reference number DRKS00034129. https://www.drks.de/search/de/trial/DRKS00034129/details.

### Dissemination policy

Changemaker updates, events and reports will be published on the project’s website https://changemaker.ki.se/ and social media channels. The trial results will be published in peer-reviewed journals. Dissemination will also take place at domestic and international conferences, educational seminars and sessions with stakeholders. The study will be disseminated in the policy community through engaging with local government representatives, civil society organisations and community leaders at all three sites.

## Discussion

### Methodological innovation

The methodological innovations of the Changemaker trial comprise the hybrid implementation-effectiveness trial design and the non-invasive assessment methods of the nutrition and health outcomes.

In health promotion and prevention, persistent gaps between research and practice are well documented. These gaps arise due to several factors including insufficient or inconsistent training,^48^ time constraints among frontline health workers,^49^ limited support for decision-making and feedback mechanisms,^49^ misaligned incentives^50^ and organisational and cultural barriers.^51^ Blended study designs that integrate clinical effectiveness research with implementation science offer strong potential to narrow these knowledge-practice gaps.^52^ Such hybrid approaches can accelerate translational gains, enhance the effectiveness of implementation strategies, and generate evidence that is more actionable for policymakers and practitioners.^21^ In SSA, the value of implementation–effectiveness trials is particularly evident given health systems often operate under high resource constraints, competing priorities and rapidly evolving public health needs. Policymakers require timely and actionable evidence that can be applied directly in real-world settings. Hybrid designs, which generate insights on both what works and how interventions can be implemented at scale, are therefore especially well-suited to contexts where rapid uptake, feasibility and sustainability are essential.^53^ The Changemaker project will efficiently generate evidence on the effect sizes and the impact pathways of a sustainable health intervention for adolescents in three emerging urban settings in SSA.

In population-based studies, objective assessments of nutrition and health outcomes are generally preferred over self-reported data because they reduce measurement error, recall bias and socially desirable responses.^54^ However, collecting biological samples for biomarker analysis may increase participant burden through added time, discomfort or perceived risk. From an ethical perspective, children and adolescents under the age of 18 years are considered a vulnerable population group, and any invasive measurements must be clinically justified and minimised to what is necessary for the study. Anthropometric examinations in population-based studies require equipment (weighing scales, stadiometer, measurement tapes) and respective logistics (transport, on-site space for equipment). In addition, z-scores need to be calculated from those measurements in relation to age- and sex-specific reference values, which need respective software or growth charts and properly trained staff. The Changemaker project employs two novel tools to measure Hb and MUACz. Both methods provide instant results, constitute objective assessments and are non-invasive. The hand-held EzeCheck photometer and the newly developed colour-coded age-specific MUACz tape (Abbott Laboratories, Chicago, IL, USA) will facilitate an optimal integration of measurement precision, ethical rigour and operational feasibility.

### Implications for public health and research

The Changemaker project will provide public health managers and authorities with access to evidence on risk behaviours related to NCDs in urban settings and how they can establish preventive environmental actions that address these risk behaviours. Such urban planning policies will contribute to enhancing food security and overall health promotion in urban SSA. The unique combination of effectiveness and process evaluation will inform decision makers about healthcare and societal costs that are preventable through sustainable health interventions in the target regions.

The Changemaker project has the potential to serve as a blueprint for implementation research on integrated health promotion for adolescents in urban areas of SSA. It will provide specific guidance to implement health interventions that decrease the risk of NCDs related to urban environments. The Changemaker project will improve the understanding of how specific interventions can be better adapted to different urban contexts and how the interventions could be scaled within and across cities. Thus, this project will contribute to optimising the implementation of sustainable health interventions.

### Trial status

May 2026, beneficiary recruitment, randomisation, baseline examinations completed and intervention is ongoing in all three countries. The trial is planned to be completed by February 2027. This protocol is version 1.1 as of 27 May 2026.

## Supplementary material

10.1136/bmjopen-2026-123282 online supplemental file 1

10.1136/bmjopen-2026-123282 online supplemental file 2

10.1136/bmjopen-2026-123282 online supplemental file 3
