# Supplementary material for: Changemaker: study protocol for sustainable adolescent health interventions to reduce non-communicable disease risks in urban Burkina Faso, Kenya and Tanzania – a hybrid type 2 cluster-randomised controlled trial
Source: BMJ Open. 2026 Oct 28;16(10):e123282. doi: 10.1136/bmjopen-2026-123282 (PMC13723846; doi:10.1136/bmjopen-2026-123282)
Supplement: Supplementary file 1 [file bmjopen-2026-123282.reviewer_comments.pdf]

PEER REVIEW HISTORY

BMJ Open publishes all reviews undertaken for accepted manuscripts. Reviewers are asked to complete a checklist review form and are provided with free text boxes to elaborate on their assessment. These free text comments are reproduced below.

ARTICLE DETAILS

Title (Provisional)

Changemaker: Study protocol for Sustainable Adolescent Health Interventions to Reduce Non-Communicable Disease Risks in Urban Burkina Faso, Kenya, and Tanzania: A Hybrid Type 2 Cluster-Randomized Controlled Trial

Authors

Sorgho, Raissa; Ferrero, Elisabetta; Shinde, Sachin; Absetz, Pilvikki; Alvesson, Helle Mölsted; Amoke, Joseph Ochieng; Linnansaari, Anu; Mlunde, Linda B; MOHIDDIN, Abdu; Muok, Erick M. O.; Ojwang, Alice Achieng; Mwanyika-Sando, Mary; Sié, Ali; Soura, Abdramane Bassiahi; Sunguya, Bruno; Temmerman, Marleen; Tinkasimile, Amani; Wamani, Arthur; Changemaker, Consortium; Annerstedt, Kristi Sidney; Danquah, Ina

---

VERSION 1 - REVIEW

**Reviewer**	**1**
**Name**	**Yamada, Masaaki**
**Affiliation**	**University of Toyama, Epidemiology and Health Policy**
**Date**	**14-Jul-2026**
**COI**	**None**

Review to the authors in BMJ Open 2026 July 14

Thank you for the opportunity of reviewing a manuscript on "Changemaker: Study protocol for Sustainable Adolescent Health Interventions to Reduce NCD Risks in Urban Burkina Faso, Kenya, and Tanzania: A Hybrid Type 2 Cluster-Randomized Controlled Trial" This study focuses on an important social issue and is a magnificent study. However, there are some issues in your protocol.

Major comments

1 Non-invasive Hb estimator seems promising in a population-based study, however, reference paper [46] does not show the validity when comparing with Hb score from the blood test.

2 Is one year enough for participants to see Hb improvements. Are there any previous reports showing Hb recovery within one year after dietary education or intervention?

3 In page 36. What is simple randomization. Did you use a dice? Or excel software?

4 In page 20 Line 390. If possible, time difference (total hours) between extra-time for nutrition and physical activity promotion in the classroom of the intervention school and the standard school-based health education program should be reported.

Minor comments

1 In page12 Line233

S of Two-folds is erased.

2 in page 35 Line 619. You wrote "Participating schools will be ~~~ in section 10." But I wonder "in section 14a" is right.

**Reviewer**	**2**
**Name**	**Khanna, Poonam**
**Affiliation**	**PGIMER, Community Medicine and School of Public Health**
**Date**	**07-Aug-2026**
**COI**	**None**

---

1. All participants provided informed consent or assent: Line no 101, this yet to be happened. somewhere authors have used future tense and some side future tense. As study yet to be conducted or started so once check with tenses in overall document. Use tenses as per requirement of the doc.

2. Sample size needs to be built on the actual primary outcome and not on a proxy outcome.

3. Line 336-338: At each study site, two schools will serve as controls, and two schools will receive the full intervention. The allocation to intervention or control arm will be done at random. As the "standard of care" package, the control arm schools will be exposed to the mass media campaign

Any specific reasons for this because this may have more chances of intervention contamination which likely to shrink the true effect size.

4. Line 582-584: The Changemaker project officially kicked off in February 2024. Baseline assessments are estimated to be conducted in Kenya in September 2025, in Burkina Faso in November 2025, and in Tanzania is January 2026.

Data collection at different points in each country across sites risks conflating seasonal effects with intervention effects. Here Timing and seasonality may confound cross-country comparison

5. Physical activity is assessed with 2 questions, WASH knowledge with 2 questions and waste management KAP 3 questions. Number of questions too less for evaluation.

6. Line 611 to 616: Loss to follow-up over an academic year is not sated into the sample size. Attrition rate needs to be mentioned and calculate sample size accordingly. Loss of follow up means school transfers, absenteeism, and family mobility.

7. Statistical power taken is 52% to 63%, authors may provide rationale for selecting such low power.

8. Authors may get the plagscan done as per my records its found to be 26% similarity index. May look for Journal Policy for plagscan similarity index.

**** The reviewer provided a marked copy with additional comments. Please contact the publisher for full details. ****

VERSION 1 - AUTHOR RESPONSE

Reviewer #1 Comments

Thank you for the opportunity of reviewing a manuscript on "Changemaker: Study protocol for Sustainable Adolescent Health Interventions to Reduce NCD Risks in Urban Burkina Faso, Kenya, and Tanzania: A Hybrid Type 2 Cluster-Randomized Controlled Trial" This study focuses on an important social issue and is a magnificent study. However, there are some issues in your protocol.

Major comments

1. **Non-invasive Hb estimator seems promising in a population-based study, however, reference paper [46] does not show the validity when comparing with Hb score from the blood test.**

Response: Thank you for this observation. We agree that reference [46] (Amrutha et al., 2024) reports test-retest reliability of the EzeCheck device rather than diagnostic validation against a laboratory blood-based haemoglobin assay. We replaced this citation with Das Mahapatra et al. (PLOS Digital Health, 2024) (now reference [48], line 492), which reports EzeCheck's diagnostic performance against blood-based reference methods in community and institutional settings, to more appropriately support the validity claim.

2. **Is one year enough for participants to see Hb improvements. Are there any previous reports showing Hb recovery within one year after dietary education or intervention?**

Response: Thanks! We agree that the timeframe for observing changes in hemoglobin (Hb) is an important consideration. A follow-up period of one year is generally considered sufficient to detect improvements in Hb concentrations resulting from dietary and nutrition-related interventions. Physiologically, hemoglobin recovery after improved iron intake typically occurs within weeks to months where iron deficiency is a contributing factor. Furthermore, several intervention studies have reported significant improvements in Hb after substantially shorter intervention periods. Systematic reviews support this timeframe: a Cochrane overview of systematic reviews found that nutrition-specific interventions, including dietary diversification, food fortification, and iron-related nutrition interventions, are associated with increased Hb

concentrations and reduced anaemia prevalence, with effects detectable well within a one-year period (da Silva Lopes et al., 2021). Additionally, a study examining the impact of nutrition education by a digital game on markers of iron-deficiency anemia among adolescent girls in Iran found that, after a 14-week intervention period, nutrition education in the intervention group significantly increased hemoglobin levels among the participants, compared to the control group (Ghadam et al., 2022).

To address this in the manuscript, we added the following text in lines 254-257: *A one-year follow-up is expected to be adequate for detecting intervention-related changes in Hb, as previous nutrition education and dietary intervention studies have reported significant improvements in hemoglobin within approximately 3-6 months [19,20].*

3. **In page 36. What is simple randomization. Did you use a dice? Or excel software**

Response: Randomization happened in different ways in the three countries. In Kenya, four identical pieces of paper, uniform in size and colour, were prepared - two marked with "YES" (indicating intervention schools) and two with "NO" (indicating control schools). Each paper was folded uniformly to conceal its contents from the school representatives. The folded papers were then placed on a flat surface and each representative randomly picked one. Schools represented by individuals who selected "YES" were designated as intervention schools, while those who selected "NO" were assigned as control schools.

In Burkina Faso, randomization was done using Stata 18 Software.

Finally, in Tanzania, the research team randomized the schools by using Microsoft Excel to allocate the schools into intervention arm and control arm:

a. The four schools were listed in excel spreadsheet.
b. A random number was assigned to each schools using the RAND function, providing a probability of numbers from 0 to 1.
c. The numbers were then copied and pasted as values in a separate column to ensure they remain constant when sorted.
d. The schools and values were then sorted from smallest to largest.
e. The first two schools (Twiga Secondary School and Mivumoni Secondary School) were allocated to the intervention arm and the last two schools (Songoro Mnyonge Secondary School and Kisauke Secondary School) were allocated to the control arm.

We added text to the manuscript in lines 649-655 to specify this process: *Schools will be randomized separately within each country using locally appropriate procedures. In Kenya, allocation was conducted through a paper-draw method, whereby representatives from each school randomly selected folded slips marked either "YES" (intervention) or "NO" (control). In Burkina Faso, schools were randomized using Stata version 18. In Tanzania, schools were assigned a random number generated in Microsoft Excel (RAND function), ranked accordingly, and allocated to either the intervention or control arm based on the randomized order.*

4. **In page 20 Line 390. If possible, time difference (total hours) between extra-time for nutrition and physical activity promotion in the classroom of the intervention school and the standard school-based health education program should be reported.**

Response: Thank you for this suggestion. We will record and report the difference in time exposure between the intervention and standard school-based health education programs as

part of the implementation evaluation, including implementation costs, which constitute one of the study's implementation outcomes.

Minor comments

5. **In page12 Line233 S of Two-folds is erased.**

Response: Thank you for noticing this typo, which we have now corrected.

6. **In page 35 Line 619. You wrote "Participating schools will be ~~~ in section 10." But I wonder "in section 14a" is right.**

Response: Thank you for pointing this out. We have now corrected this sentence by adding the following text to lines 629-630: *Participating schools will be selected based on the criteria described in the "Eligibility criteria" section.*

Reviewer #2 Comments

1. **All participants provided informed consent or assent: Line no 101, this yet to be happened. somewhere authors have used future tense and some side future tense. As study yet to be conducted or started so once check with tenses in overall document. Use tenses as per requirement of the doc.**

   Response: Thank you. We revised the manuscript to ensure that the future tense is used throughout, except when the present tense is most appropriate.

2. **Sample size needs to be built on the actual primary outcome and not on a proxy outcome.**

Response: We have now inserted the sample size calculation based on the piloting data for MUAC z-score measurements. The manuscript reads as follows: *Our sample size calculation was based on the primary effectiveness outcome of MUAC z-score. The MUAC z-score tape is a novel tool that has not been used in previous intervention studies. Therefore, we relied on unpublished piloting data of variations in MUAC z-score in the target populations. The observed means ranged from –0.32 in girls to –1.06 in boys, each with a standard deviation of 1.*

3. **Line 336-338: At each study site, two schools will serve as controls, and two schools will receive the full intervention. The allocation to intervention or control arm will be done at random. As the "standard of care" package, the control arm schools will be exposed to the mass media campaign. Any specific reasons for this because this may have more chances of intervention contamination which likely to shrink the true effect size.**

Response: We acknowledge the concern regarding potential contamination from the mass media campaign. However, we considered it ethically important to provide this component to both intervention and control schools, as mass media campaigns promoting healthy diets and physical activity are included among the WHO-recommended "Best Buys" (i.e., a minimal, low-cost, population-level intervention already recommended for universal implementation regardless of trial participation) and other evidence-based interventions for the prevention of noncommunicable diseases. Providing access to these broader health promotion messages to all participating schools ensures that no group is denied exposure to established public health recommendations. While some contamination may occur and could reduce the observed effect

size, the additional school-based intervention components delivered only in intervention schools are expected to generate measurable differences between study arms.

Design-wise, using the mass media campaign as the "standard of care" comparator allows the trial to estimate the *incremental* effect of the full Changemaker intervention (school gardening, classroom education, and motivational health talks) above and beyond a minimal, scalable, already-recommended intervention, which is the policy-relevant question, since mass media campaigns alone are already being scaled in these settings independent of this trial. We agree this is a more conservative test than a true no-intervention control and will likely attenuate the observed effect size relative to a null comparator; this was accounted for in our sample size calculation, which is based on detecting the *additional* effect of the multi-component intervention over this active comparator, not the absolute effect of Changemaker versus no intervention at all.

We also note that this is a designed common exposure across arms rather than contamination in the conventional sense (i.e., unintended cross-arm exposure to gardening, classroom, or motivational-talk components), which remains controlled for through school-level cluster randomization and geographic separation of intervention and control schools within each site.

To reflect this in the manuscript, we added the following text to lines 342-346: *This approach was adopted for ethical reasons, as mass media interventions are recommended as population-level strategies for promoting healthy diets and physical activity. The intervention effect will therefore be evaluated based on the added value of the school-based components delivered exclusively in intervention schools.*

4. **Line 582-584: The Changemaker project officially kicked off in February 2024. Baseline assessments are estimated to be conducted in Kenya in September 2025, in Burkina Faso in November 2025, and in Tanzania is January 2026. Data collection at different points in each country across sites risks conflating seasonal effects with intervention effects. Here Timing and seasonality may confound cross-country comparison**

Response: We appreciate this important observation. The timing of baseline and endline assessments was determined by the school calendars and implementation realities in each participating country, making simultaneous data collection across all sites not feasible. To minimize the potential influence of seasonality, baseline and endline assessments within each country will be conducted during comparable periods of the school year. In addition, country and time-related factors will be accounted for in the statistical analyses through appropriate fixed effects and adjustment variables. This approach will help reduce the risk that seasonal variation influences intervention effects and will strengthen the validity of cross-country comparisons.

We added the following text to the manuscript (lines 592-596): *Baseline and endline data collection schedules are aligned with the academic calendars of each participating country. Although assessments will take place at different calendar times across countries, data collection within each country will occur during comparable school-year periods. Statistical models will further adjust for country- and time-specific effects to account for potential seasonality and contextual differences.*

5. **Physical activity is assessed with 2 questions, WASH knowledge with 2 questions and waste management KAP 3 questions. Number of questions too less for evaluation.**

Response: The selection of these measures reflected a deliberate decision to minimize participant burden. The overall data collection package is extensive, and we aimed to minimize disruption to the school day and avoid participant fatigue among adolescents. The questions included are standardized and have been previously validated for use in similar settings. Additionally, findings from the pilot study showed that completion of the survey already required over one hour, and adding additional items would have substantially increased respondent burden. These measures are secondary rather than primary study outcomes, and therefore brief validated instruments were considered appropriate for the study objectives.

6. **Line 611 to 616: Loss to follow-up over an academic year is not sated into the sample size. Attrition rate needs to be mentioned and calculate sample size accordingly. Loss of follow up means school transfers, absenteeism, and family mobility**.

Response: Thank you for this important observation. As described in comment #2, we have now based the revised sample size calculation on unpublished piloting data for MUAC z-score and are presenting the expectations for loss to follow-up. We have updated the manuscript as follows:

For this trial, we will enroll adolescents in 12 different schools, representing six clusters per intervention arm. The intra-class correlation coefficient within schools was estimated at 0.025 [51], representing moderate heterogeneity. Based on differences in mean MUAC z-score observed during piloting and assuming a standard deviation (SD) of 1, the sample size of 154 adolescents per school will enable us to detect between-group differences in mean MUAC z-score of 0.3 or larger with statistical power (1-β) of 80% and an α-level of 0.05 (two-sided). To account for 20% loss to follow-up, we aimed at 190 participants per school.

7. **Statistical power taken is 52% to 63%, authors may provide rationale for selecting such low power.**

Response: We thank the reviewer for raising this point. At the time of the original power calculation, no empirical variance estimates for MUAC z-score were available for this population, and the initial calculation relied on conservative assumptions from the literature, which resulted in the low power reported (52–63%). Subsequent piloting in the target population provided site-specific estimates of the standard deviation of MUAC z-score, allowing us to revise the sample size calculation using empirically grounded parameters. Based on these updated estimates, a sample size of n=154 per school achieves 80% power (1-ß = 0.80) to detect the pre-specified effect size at α = 0.05.

8. **Authors may get the plagscan done as per my records it's found to be 26% similarity index. May look for Journal Policy for plagscan similarity index. Look into this.**

Response: Thank you for this suggestion. The manuscript has already undergone similarity screening through BMJ's standard editorial processes using Turnitin, and no concerns were raised regarding text similarity. We have nevertheless carefully reviewed the manuscript to ensure appropriate citation of sources and adherence to the journal's publication and originality requirements.
