# Supplementary material for: Changemaker: study protocol for sustainable adolescent health interventions to reduce non-communicable disease risks in urban Burkina Faso, Kenya and Tanzania – a hybrid type 2 cluster-randomised controlled trial
Source: BMJ Open. 2026 Oct 28;16(10):e123282. doi: 10.1136/bmjopen-2026-123282 (PMC13723846; doi:10.1136/bmjopen-2026-123282)
Supplement: Supplementary file 3 [file bmjopen-16-10-s002.docx]

**Participant Information for Adolescents**

– Version 1.2, 01 November 2024 –

CHANGEMAKER – TRIAL

Promoting co-designed sustainable health interventions with young changemakers for reduced risk of non-communicable diseases (NCDs) in urban Burkina Faso, Kenya and Tanzania

Principal Investigators:

- Prof. Ina Danquah, Hertz-Chair Innovation for Planetary Health and Director at Center for Development Research (ZEF), University of Bonn, Genscherallee 3, 53113 Bonn, Germany, Phone: +49 228 73 1970
- [local PIs]

Dear Madam, Dear Sir,

Thanks for taking the time to read this text. In order to improve the prevention of diseases, we want to better understand how they can be avoided. For this purpose, we are inviting you to an experimental study. During this study, we will ask you to participate in a school programme, collect data from you, and compile the data in a database. This study has been initiated by the University of Bonn in Germany and by Karolinska Institute in Sweden. It is sponsored by the European Union, and the principal investigators in your country are [local PIs and institutions]. Before you can decide about your participation, you need to know the nature, significance and scope of this project, as well as the risks and potential benefits. Please read the following information carefully. If you have any questions or need further information, please contact us. **Take your time and make your decision calmly.**

Your participation in this study is voluntary. You have the right – without giving reasons – to cancel your participation. This will not affect your standard medical care in any way. You can only participate when your guardians also agree.

**What are the goals of the study?**

In this study, we collect data from you in interviews and observations. We and other researchers will work with the data to understand better how diseases develop and how we can prevent this. You can find more information on our project website: https://cordis.europa.eu/project/id/101137359

**What are the tasks and the data?**

The present project consists of an **intervention package and data collection** to identify the effects of the intervention strategies.

**Intervention package:**

We will enrol adolescents between 14 and 16 years of age in 4 different schools in your city. Two schools will receive the intervention package and two schools will receive social media information only. In each school, we will enrol 190 adolescents. Whether your school will be an intervention school or a control school will be determined completely at random / by chance. This means, you may or may not participate in the intervention. You will definitely participate in the data collection. The same procedures will be followed in two other cities in sub-Saharan Africa.

Over the period of one academic year, the intervention includes:

i) School-based gardening, nutrition and health education for teenagers, and face-to-face health talks with Community Health Volunteers/Workers/Promoters;
ii) Home gardening at your household, and
iii) Mass media approaches, e.g., social media. The mass media component includes the following messages on healthy lifestyles:

• Reduce overall energy consumption,

• Reduce salt/sodium intake,

• Reduce intake of trans-fats from industrialized products,

• Reduce intake of sugar-sweetened beverages,

• Increase intake of fruits and vegetables,

• Enhance education on healthy nutrition and physical activity,

• Promote physical activity on individual and community levels.

**Data collection:**

The data collection will be done twice: i) before the intervention package will be rolled out and ii) when the intervention has finished (after one academic year). The data collection procedures will take no longer than 1 hour. They will be performed by experienced and well-trained team members in a private space at your school. All data, which we collect in this study, will be treated confidentially and will be pseudonymised. This means that only code numbers and no names will be used in the analysis. The data will be collected in interviews and in physical examinations. We will **not collect** any biological samples from you.

The **interviews** will cover the following topics:

• Your dietary practices,

• Your knowledge about healthy diets,

• Your physical activity,

• Your knowledge and practices about hygiene, vegetable gardening, waste management,

• Your family’s socio-economic background, e.g., your parents’ educational level, number of siblings,

• Your mental and physical wellbeing.

The **physical examinations** will comprise the following measurements:

• mid-upper arm circumference,

• haemoglobin concentration (red blood pigment) through a smartphone-based app (no blood collection).

**How will my data be used?**

The General Data Protection Regulation (EU-DSGVO, Art. 6 Par. 1 lit. a) and Art 9 Para. 2 lit. a)) forms the legal basis of collecting, processing, storing, and analysing your data. The person responsible for processing your data is [local PIs and institution]. The data obtained from you will be used to better understand how chronic diseases, like sugar disease, high blood pressure, and overweight can be prevented. For this purpose, we will store, share and analyse your research data. The data will be stored for a maximum duration of 10 years after the end of the study. **We will not collect any body material from you.**

**What are potential risks?**

Some people can feel that the following is unpleasant or a burden:

- the interview takes one hour;
- the interview includes personal topics such as dietary practices and wellbeing;
- the participants have to wear light clothes during anthropometric measurements (mid-upper arm circumference);
- the notification of findings which, e.g., suggest anaemia (low haemoglobin).

We collect data from you. We do everything we can to keep your data protected and away from misuse. However, we cannot fully rule out that other people could find your data. This risk increases when you share personal information in the internet or on social media. Below, we give you more information how we protect your data.

**What are potential benefits?**

You will benefit directly from this study. Through participation in the activities described above, we expect that you improve your nutritional behaviour, enhance your physical activity, and follow a healthier lifestyle. The measurements we will undertake during the physical examination will be promptly explained to you. If needed, we will inform your parents, so that they can seek additional care and/or counselling. Moreover, the information derived from this study can help to better understand how we can prevent diseases among adolescents in your city and in other sub-Saharan African countries.

**Do I have a financial benefit?**

You do not have to pay anything for participating in this study. We will have small gifts for you to compensate for your time and will provide refreshments during the workshops and data collection sessions.

**How will my data be protected?**

Your research data (interview information, observations, body weight etc.) will not be connected with your personal information (name, birth date, address), but will be connected with a code number (e.g., „XG72jdk784“). Your personal information will remain with [local institution] and will be separately stored from your research data. Your data will be stored as password-protected and encrypted files on institutional secure cloud-based servers. Your personal information will not be given to people outside of this study team. This means, nobody who works with your research data will see your name, birth date or address. Also, when we publish the study’s findings, your name will not be mentioned.

Your research data will be given to people outside of this study team only with the assigned code number. Your parents have received more information about this.

**Can I change my opinion?**

Your participation is totally voluntary. You can withdraw your assent at any time in writing or verbally without giving reasons and without any disadvantages for you. If you withdraw your assent, no further data will be collected. You can then decide if you want the research data to remain in our database or if all previously collected data should be deleted. If you want to withdraw, please contact the persons listed on the bottom of this form. You parents can also do this for you.

**What are my additional rights in relation to data protection?**

We can only store your personal information and your research data when you agree to this. The person who is responsible for data protection is:

Data manager at study centre: [contact details]

Data protection officer of the principal investigator’s institution:

Eckehard Wesemann, Genscherallee 3, 53113 Bonn, Germany

Tel.: +49 228 73 1234, datenschutz@uni-bonn.de

From this person, you can request information about your data, correction of wrong data, deletion of your data, and limiting the use of your data.

If you have any complaints about your data handling, you can reach out to the data protection supervisory authority.

Data protection supervisory authority of your state: [contact details]

Data protection supervisory authority responsible for the principal investigator:

Landesbeauftragte für Datenschutz und Informationsfreiheit Nordrhein-Westfalen,

Postbox 20 04 44, 40102 Düsseldorf, Germany, Tel.: +49 211 38424 0, poststelle@ldi.nrw.de

**From who can I get more information?**

If you have any questions about this study, please contact:

- Prof. Ina Danquah, Hertz-Chair Innovation for Planetary Health and Director at Center for Development Research (ZEF), University of Bonn, Genscherallee 3, 53113 Bonn, Germany, Phone: +49 228 73 1970
- [local PIs]

Prof. Ina Danquah, Principal Investigator	[signature local PI]

Bonn, 01 November 2024
