# Supplementary material for: Changemaker: study protocol for sustainable adolescent health interventions to reduce non-communicable disease risks in urban Burkina Faso, Kenya and Tanzania – a hybrid type 2 cluster-randomised controlled trial
Source: BMJ Open. 2026 Oct 28;16(10):e123282. doi: 10.1136/bmjopen-2026-123282 (PMC13723846; doi:10.1136/bmjopen-2026-123282)
Supplement: Supplementary file 4 [file bmjopen-16-10-s003.docx]

**Participant Information for Guardians**

– Version 1.2, 01 November 2024 –

CHANGEMAKER – TRIAL

Promoting co-designed sustainable health interventions with young changemakers for reduced risk of non-communicable diseases (NCDs) in urban Burkina Faso, Kenya and Tanzania

Principal Investigators:

- Prof. Ina Danquah, Hertz-Chair Innovation for Planetary Health and Director at Center for Development Research (ZEF), University of Bonn, Genscherallee 3, 53113 Bonn, Germany, Phone: +49 228 73 1970
- [local PIs]

Dear guardian(s),

Your child has been invited to participate in an experimental study. This study has been initiated by the University of Bonn in Germany and by Karolinska Institute in Sweden. It is sponsored by the European Union, and the principal investigators in your country are [local PIs and institutions]. Before you can decide about this invitation, you need to know the nature, significance and scope of this project, as well as the risks and potential benefits. Please read the following information carefully. If you have any questions or need further information, please contact us. Take your time and make your decision calmly.

**Voluntariness**

Participation in this study is voluntary. You have the right – without giving reasons – to disagree with your child’s participation in this study, to cancel your given consent, to cancel your given consent to the analysis of your child’s data, to ask for deletion of the given data, and to terminate the study participation without affecting your child’s and your own standard medical care in any way.

**Purpose of the Study**

Unhealthy lifestyles are the main risk factors for the development of so-called non-communicable diseases (NCDs). Lifestyle factors are dietary practices, physical activity, smoking, alcohol consumption, screen time, sleeping rhythm and others. Unhealthy lifestyles are connected with the development of overweight and obesity – this is when a person has too much weight for his/her height. When lifestyles are unhealthy, this can lead to overweight and obesity, and also the development of NCDs, including sugar disease (type 2 diabetes), high blood pressure (hypertension), high blood lipids (dyslipidemia), stroke, heart attack, atherosclerosis (cardio-vascular diseases), and cancers.

The reasons for unhealthy lifestyles can vary from person to person. They are a mix of factors on different levels: individual, family and friends, neighborhood, health system, and legal frameworks. Adolescence is an important age for the establishment of lifestyles. Therefore, through this study we would like to understand to what extend a package of different intervention strategies at different levels can promote healthy lifestyles and reduce the risk of NCDs among adolescents.

**Course of the Study**

Preceding the study, you are asked to read this participant information and to sign the informed consent form. Your child will be asked to sign an informed assent form, too. The present project consists of an **intervention package and data collection** to identify the effects of the intervention strategies.

**Intervention package:**

We will enrol adolescents between 14 and 16 years of age in 4 different schools in your city. Two schools will receive the intervention package and two schools will receive social media information only. In each school, we will enrol 190 adolescents. Whether your child’s school will be an intervention school or a control school will be determined completely at random / by chance. This means, your child may or may not participate in the intervention. Your child will definitely participate in the data collection. The same procedures will be followed in two other cities in sub-Saharan Africa.

Over the period of one academic year, the intervention includes:

i) School-based gardening, nutrition and health education for teenagers, and face-to-face health talks with Community Health Volunteers/Workers/Promoters;
ii) Home gardening at the child’s household, and
iii) Mass media approaches, e.g., social media. The mass media component includes the following messages on healthy lifestyles:

• Reduce overall energy consumption,

• Reduce salt/sodium intake,

• Reduce intake of trans-fats from industrialized products,

• Reduce intake of sugar-sweetened beverages,

• Increase intake of fruits and vegetables,

• Enhance education on healthy nutrition and physical activity,

• Promote physical activity on individual and community levels.

**Data collection:**

The data collection will be done twice: i) before the intervention package will be rolled out and ii) when the intervention has finished (after one academic year). The data collection procedures will take no longer than 1 hour. They will be performed by experienced and well-trained team members in a private space at your child’s school. All data, which we collect in this study, will be treated confidentially and will be pseudonymised. This means that only code numbers and no names will be used in the analysis. The data will be collected in interviews and in physical examinations. We will **not collect** any biological samples from your child.

The **interviews** will cover the following topics:

• Your child’s dietary practices,

• Your child’s knowledge about healthy diets,

• Your child’s physical activity,

• Your child’s knowledge and practices about hygiene, vegetable gardening, waste management,

• Your family’s socio-economic background, e.g., your own educational level, number of siblings,

• Your child’s mental and physical wellbeing.

The **physical examinations** on your child will comprise the following measurements:

• mid-upper arm circumference,

• haemoglobin concentration (red blood pigment) through a smartphone-based app (no blood collection).

**Data Usage**

The General Data Protection Regulation (EU-DSGVO, Art. 6 Par. 1 lit. a) and Art 9 Para. 2 lit. a)) forms the legal basis of collecting, processing, storing, and analysing your data. The person responsible for processing your data is [local PIs and institution]. Your child’s pseudonymised data (i.e., only with a code number) will be transferred via password-protected and secure cloud servers to the coordinating institution of the study for the purpose of processing, storage, and analysis. The data will be stored for a maximum of 10 years after the end of the study. That is:

- Prof. Ina Danquah, Hertz-Chair Innovation for Planetary Health and Director at Center for Development Research (ZEF), University of Bonn, Genscherallee 3, 53113 Bonn, Germany, Phone: +49 228 73 1970

**Potential Risks**

Some study participants can feel that the following is unpleasant or a burden:

- the interview takes one hour;
- the interview includes personal topics such as dietary practices and wellbeing;
- the participants have to wear light clothes during anthropometric measurements (mid-upper arm circumference);
- the notification of findings which, e.g., suggest anaemia (low haemoglobin).

**Potential Benefits**

Adolescents who participate in the intervention will benefit directly from this study. Through participation in the activities described above, we expect that your child improves his/her nutritional behaviour, enhances his/her physical activity, and follows a healthier lifestyle. The measurements we will undertake during the physical examination will be promptly explained to your child. If needed, we will refer your child to seek additional care and/or counselling. Moreover, the information derived from this study can help to inform efforts to improve adolescent nutrition and health in other schools of your city and in other sub-Saharan African countries.

**Insurance**

For formal reasons, we point out that no specific insurance has been contracted for study participants. Rather, the principal investigators are insured against liability claims which arise from culpable performance by the employer's liability insurance.

**Data Protection**

By signing the informed consent form you declare that you agree that the principal investigator and team members collect and process your child’s personal data for the purpose of the above-mentioned analyses (Article 6(1)(a) GDPR and Article 9(2)(a) GDPR).

The person responsible for data processing is:

[local data manager and contact details of the institution]

The data will be treated confidential at all times. The data will be forwarded in pseudonymised form to [local PI] and by bodies commissioned by him/her for the purpose of scientific evaluation. Only the responsible persons in the respective study centre have access to your child’s personal data.

Pseudonymised means that no information that can be used to identify your child directly (e.g., name, contact information, date of birth, etc.) will be used, but only a number and/or letter code. The investigator will create a pseudonymisation list, which will be stored separately at the study centre and is subject to technical and organisational measures to ensure that personal data cannot be assigned to you by unauthorised persons. Decryption will only take place when you request to delete your child’s data.

The data will be stored for 10 years after completion or cancellation of the study. They are secured against unauthorised access. They will be deleted when they are no longer needed for the scientific purposes. They will be deleted after 10 years at the latest.

**Risks Associated with Data Processing**

Any collection, storage, utilisation, and transmission of data involves confidentiality risks (e.g., the possibility of identifying the person concerned). These risks cannot be completely ruled out and increase the more data can be linked together. The principal investigators of the study ensure you that they will do everything possible to protect your child’s and your own privacy according to the state of the art and will only pass on data to organisations that can demonstrated a suitable data protection concept. There are no medical risks associated with data processing.

The data may also be transferred to countries outside of sub-Saharan Africa and the EU, e.g., the United States of America (USA). These countries may have a lower level of data protection. With your consent, you agree that the data may also be transferred to those countries.

The principal investigators of this study will endeavour to ensure an appropriate level of data protection. Please note: The data will only be passed on in pseudonymised form. The code (the pseudonym) can only be decrypted within the EU or within the study centre in order to assign the pseudonymised data to your child. The following protection measures are also in place: data will only be transferred on password-protected files and all study team members are instructed on full confidentiality. You can obtain a copy of these safeguards and access them at the following address:

[local data manager].

**Withdrawal of Consent**

You can withdraw your consent at any time in writing or verbally without giving reasons and without any disadvantages for you and your child. If you withdraw your consent, no further data will be collected. However, the data processing carried out up to the point of withdrawal remains lawful.

In the event of withdrawal, you can also request deletion of your child’s data. In that case, we have to link the pseudonymised data with your child’s personal information, before deletion.

**Your Rights in Relation to Data Protection**

You have the right to request information from the responsible person at your study centre about your child’s personal data that we have stored (including a provision of a copy of the data free of charge). You can also request the correction of inaccurate data and, if necessary, the transfer of the data your child has provided and the restriction of its processing.

In general, please contact your study centre, because only the study centre can fully access your data or provide the corresponding information due to the pseudonymisation process. Against this background, the principal investigators of the study can only help to a limited extent.

If you have any concerns regarding data processing and compliance with data protection requirements, you can also contact the following data manager:

Data manager at study centre: [contact details]

Data protection officer of the principal investigator’s institution:

Eckhard Wesemann, Genscherallee 3, 53113 Bonn, Germany

Tel.: +49 228 73 1234, datenschutz@uni-bonn.de

In addition, you have the right to lodge a complaint with a data protection supervisory authority. If you have any concerns regarding the handling of your personal data, you can contact the following organisations:

Data protection supervisory authority of your state: [contact details]

Data protection supervisory authority responsible for the principal investigator:

Landesbeauftragte für Datenschutz und Informationsfreiheit Nordrhein-Westfalen,

Postbox 20 04 44, 40102 Düsseldorf, Germany, Tel.: +49 211 38424 0, poststelle@ldi.nrw.de

A list of all competent data protection supervisory authorities in the European Union can be found here: https://www.bfdi.bund.de/DE/Infothek/Anschriften_Links/anschriften_links-node.html

**Honorarium and Costs**

Your child or you do not have to pay anything for participating in this study. We will have small gifts for you to compensate for your time and transportation costs (if any). Also, we will provide refreshments during the workshops and data collection sessions.

**Contact for Questions**

If you have any questions about this study, please contact:

- Prof. Ina Danquah, Hertz-Chair Innovation for Planetary Health and Director at Center for Development Research (ZEF), University of Bonn, Genscherallee 3, 53113 Bonn, Germany, Phone: +49 228 73 1970
- [local PIs]

Prof. Ina Danquah, Principal Investigator	[signature local PI]

Bonn, 01 November 2024
